# Material science lesson from the biological photosystem

**DOI:** 10.1186/s40580-016-0079-5

**Published:** 2016-08-15

**Authors:** Younghye Kim, Jun Ho Lee, Heonjin Ha, Sang Won Im, Ki Tae Nam

**Affiliations:** Department of Materials Science and Engineering, Seoul National University, 151-744 Seoul, Korea

**Keywords:** Porphyrin, Thylakoid Membrane, Carbon Fixation, Forster Resonance Energy Transfer, BiVO4

## Abstract

Inspired by photosynthesis, artificial systems for a sustainable energy supply are being designed. Each sequential energy conversion process from light to biomass in natural photosynthesis is a valuable model for an energy collection, transport and conversion system. Notwithstanding the numerous lessons of nature that provide inspiration for new developments, the features of natural photosynthesis need to be reengineered to meet man’s demands. This review describes recent strategies toward adapting key lessons from natural photosynthesis to artificial systems. We focus on the underlying material science in photosynthesis that combines photosystems as pivotal functional materials and a range of materials into an integrated system. Finally, a perspective on the future development of photosynthesis mimetic energy systems is proposed.

## Introduction

Photosynthesis is a profound source of inspiration for energy transport, conversion and storage systems. It is the only existing light-driven growth process in nature and converts 4.2 × 10^17^ kJ of energy into biomass per year. As man has faced an energy crisis rooted in a reliance on fossil fuels, photosynthesis-inspired energy systems have been developed. Each sequential process in photosynthesis, from the light collection in the antenna to the synthesis of sugar from carbon dioxide, is a constructive model for renewable energy development. However, though it is a fully effective system for the metabolism of organisms in a biological environment, the light to sugar conversion efficiency of 5 % is very low as a commercial device, and protein itself is a precarious material to directly apply in an artificial system. Thus, biological photosynthesis should be adapted for new energy applications by reengineering the system based on the key lessons of nature.

**Fig. 1:**
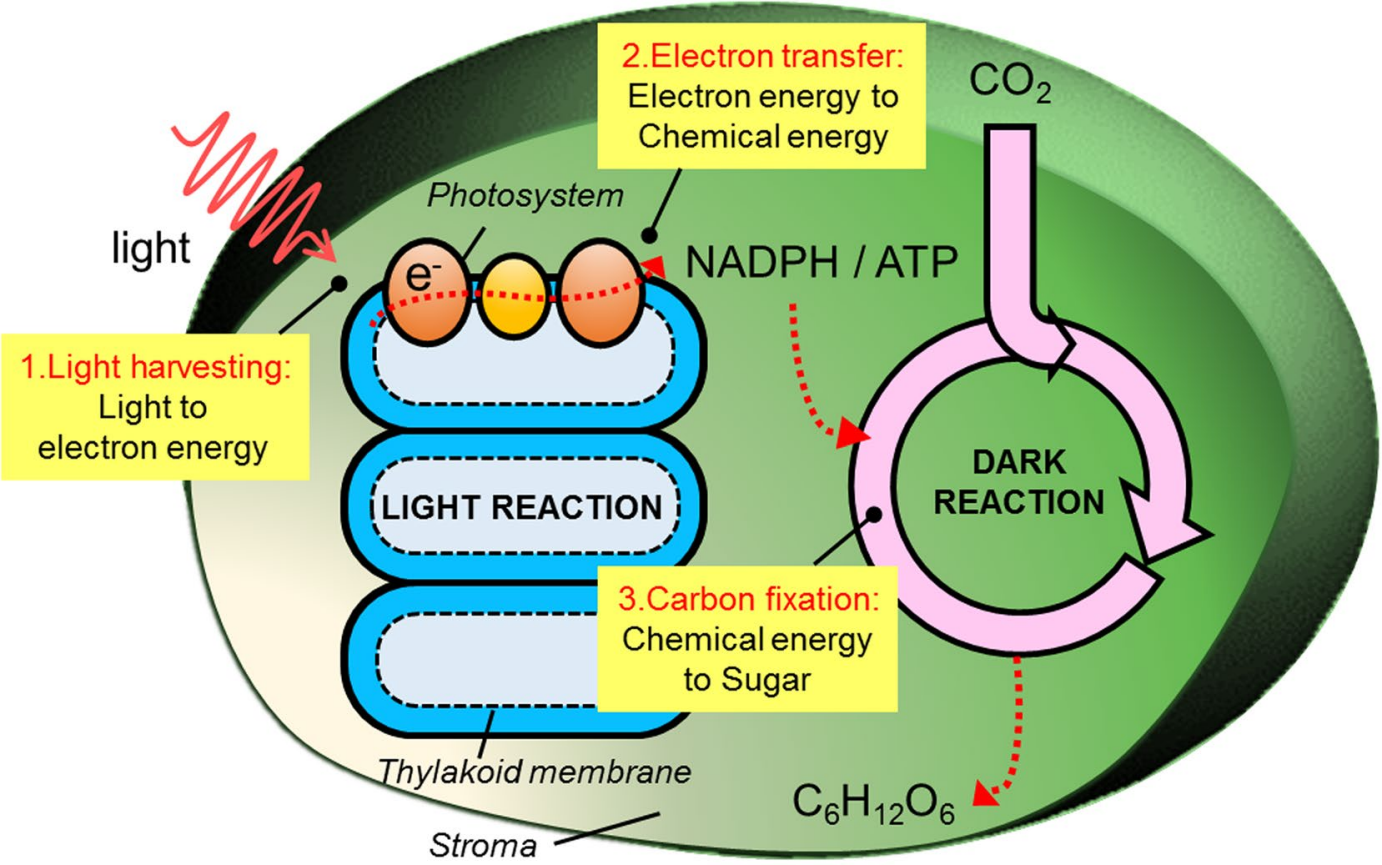
Energy conversion steps in oxygenic photosynthesis

In oxygenic photosynthesis, which takes place in plants and some bacteria, water and carbon dioxide are converted into sugar and oxygen under sunlight (6CO_2_ + 6H_2_O + light → C_6_H_12_O_6_ + 6O_2_). From the perspective of an energy cycle, photosynthesis can be divided into three energy conversion processes: light harvesting, electron transfer, and carbon fixation. Figure 1 shows the concise scheme of each process in photosynthesis. Through these sequential reactions, the overall photosynthesis can be described as the process of energy conversion from light, through electron energy and chemical energy, to carbohydrate biomass. In a plant, the chloroplast is the base organelle that conducts net photosynthesis. Its interior consists of piled thylakoid membranes in which the photosynthetic proteins that participate in the light reaction are tightly embedded, and the exterior is called the stroma, where the dark reaction proceeds.

Photosynthesis consists of various reaction stages carried over through several energetic and electronic interactions. Thus, each system that handles a specific reaction should be designed to collaborate inside the network. The photosystem is surrounded by a light-harvesting complex in the thylakoid membrane to effectively accept absorbed light. Moreover, its electron-emitting direction is directed toward the stromal side to facilitate the direct use of the chemical product in the dark reaction in the stroma. Nature has also evolved to optimize its system to operate the reactions in the given condition. Photosynthesis is sensitively regulated by various conditions on earth, and the structure of the system is continually reconstructed. In this way, nature acquires a sophisticated design skill using the tools of biomaterials, mostly photosynthetic proteins, and provides significant lessons for various energy systems.

Various approaches to mimic or modify natural photosynthesis have been developed. Protein is a desirable functional material as a catalyst or electron/energy carrier but requires particular handling. In photosynthesis mimetic research, developing a practical biomaterial based on a protein or peptide has been attempted [1]. Furthermore, the materials should be integrated into an artificial system that is mostly constructed in the hybrid form of organic/inorganics [2, 3]. In this review, the key lessons from photosynthesis are classified into three energy conversion steps: (1) light harvesting, (2) electron transfer, and (3) carbon fixation. Based on the underlying science developed from the natural system, novel strategies to realize a photosynthesis-inspired energy system are presented together with an introduction to recently demonstrated examples.

## Light harvesting and energy transfer

Light harvesting is the very first process that directly leads to charge separation at the reaction center in photosystems. The antenna of the photosynthetic system has evolved to absorb sufficient sunlight and effectively concentrate the collected energy in the reaction center. The ingeniously arranged natural pigments supported by a large protein complex teach us an essential lesson for a well-made light harvesting system.

### Antenna system in natural photosynthesis

In nature, the overall light harvesting takes place in a light-harvesting complex that is mainly composed of organic pigments. Because the main output of solar irradiance is in the visible and near IR region, the absorbance spectrum of photosynthetic pigments mostly falls within this range. Nature has chosen chlorophyll as a prime building block of the light-harvesting complex, and it exhibits optimized absorption properties for the solar spectrum, supported by other accessory pigments. The pigments collectively assemble into the light-harvesting complex and ultimately play a role as an antenna, transferring energy to the reaction center to induce charge separation.

Chlorophyll is the major photosynthetic pigment in the natural light harvesting complex. It has a porphyrin ring with a magnesium ion at its center, and its absorption spectrum is tuned depending on the substituents of the structure and the chemical bond saturation. Chlorophyll a is the main component in universal organisms, and it also acts as a primary donor pair for the reaction center of photosystems. The absorption range of chlorophyll encompasses most of the spectrum of visible light but shows an absence of absorption between 500 and 600 nm, which makes the pigment green. This ‘green gap’ is filled by other accessory pigments such as carotenoids and phycobilins [4].

When the pigments reach the excitation state by absorption, the excited energy is immediately transferred to other adjacent pigments and ultimately collected in the reaction center. To retain the optimal energy pathway, the pigment molecules are positioned to keep a proper arrangement with nearby molecules. The classical mechanism for excited energy transfer is based on Forster resonance energy transfer (FRET), which is derived from electric dipole–dipole interactions between molecules. Herein, an exciton from a donor molecule hops to an adjacent acceptor at a speed proportional to R^6^ (R, distance between two pigments). However, recent studies have repeatedly raised the objection that the extremely fast energy transfer cannot rely solely on FRET. Additionally, Engel et al. observed crucial evidence of quantum coherence for the energy transfer in the antenna of green sulfur bacteria in 2007 [5]. Since then, quantum coherence has been suggested as a rational strategy for efficient energy transfer in the photosynthetic antenna. Coherently oscillating excitons can travel over molecules as a huge wave and provide very fast energy transfer in less than 1 ps [6]. There is still a need to clarify the energy transfer mechanism in the photosynthetic antenna, but the distinct lesson is that three-dimensional arrangement of the pigments regulates the intermolecular force that controls the net energy transfer.

How, then, are the pigments stably fixed into the desired arrangement in biological systems? Protein is the template of the antenna that supports the pigments in the complex. Integrated with protein, chlorophylls maintain an average neighbor distance of 1 nm and form a particular shape and size of the light-harvesting complex that avoids quenching but facilitates energy transfer [7]. The protein scaffold also dominates the net absorption wavelength. By organizing the pigments diversely with a specific protein scaffold, the absorption wavelength can be easily tuned, and this is one of the survival strategies of photosynthetic organisms to secure sufficient light in varying situations. The branched residues of the protein scaffold can directly interact with pigments by the formation of hydrogen bonds or the stabilization of the pigment [8]. Thus, the interaction of the proteins and the pigments is the main factor in the construction of the light-harvesting complex in nature.

### Artificial antenna for light harvesting

Inspired by the photosynthetic antenna system, an artificial light harvesting system has been developed using various approaches. Among the interesting features discovered in nature, two strategies developed through evolution have incentivized the design of novel light-harvesting systems: (1) tuning the optical properties of the antenna by organizing the chromophore molecules in a particular arrangement and (2) engineering the structure of the light-harvesting complex to enable effective energy transfer toward the reaction center. The following are some previous works applying lessons from the natural antenna.

#### Chromophore assembly

In the design of the light-harvesting complex, selecting a proper chromophore or combination of chromophores primarily determines the absorption property. Porphyrin dye is the typical chromophore used in engineered light-harvesting complexes in which chlorophyll is also included. It has a pyrrole subunit connected to a heterocyclic structure, usually with a metal ion inserted at the center of the ring. In addition to porphyrin, various chromophores are utilized according to the given situation, as the natural antenna uses some carotenoids to fill the green gap of chlorophylls. Then, to fabricate the net assembly as a light-harvesting complex, an alternative to substitute for the protein support in nature is required. First, introducing a manufactured organic and/or inorganic template is a promising strategy to tighten the chromophores into the desired structure. The self-assembly of chromophores can also be induced by providing particular conditions without needing to actively introduce the molecule into the scaffold. The two methods can be synergized by appropriate simultaneous utilization.

A metal template can be a stable framework for the chromophores in artificial light-harvesting systems. Furumaki et al. reported the formation of bacteriochlorophyll aggregates on gold nanorods in 2014 [9]. The bacterial chromophores could be finely aggregated by forming hydrogen bonds to the hydroxylated gold surface. The observed spectroscopic properties were similar to those of the natural bacterial light-harvesting complex, despite the difference in the mesoscopic structure. In another approach, Grill et al. utilized a gold surface as an activation template throughout the chromophore assembly process [10]. Herein, porphyrin formed a covalently bound molecular nanostructure on the gold surface that provides an essential support for the porphyrins to be connected.

An organic template including peptides, organic molecules, and polymers can also efficiently stabilize chromophores. In the artificial antenna, covalent bonding on the organic scaffold can be used to fix the chromophores in the desired arrangement, whereas the natural chromophores interact with protein residues mainly by dipole interactions or hydrogen bonds. In 2013, Kang et al. introduced a helical peptoid for the fine regulation of the porphyrin arrangement [11]. On a peptoid scaffold, several porphyrins can be conjugated in a specific arrangement during the peptoid synthesis step. The intermolecular distance, orientation and number of porphyrins were easily controlled by arranging the desired sequence. This approach presents a practical method to regulate an elaborate structure on the molecular scale.

**Fig. 2:**
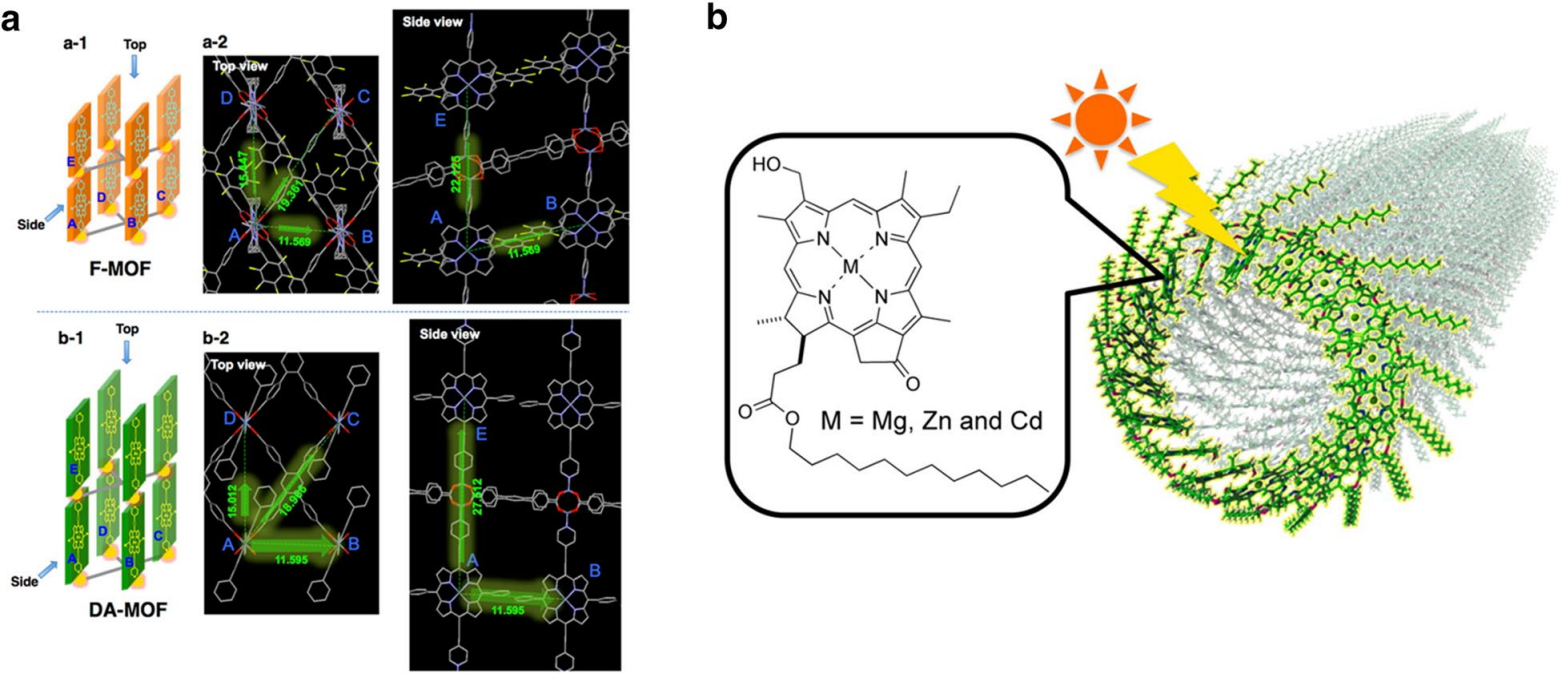
Schematic representation of artificial antenna. **a** Porphyrin based MOFs. **b** Nanotube constructed by synthetic metal chlorophyll derivatives Adapted with permission from ref [14] and [19], Copyright 2013 and 2016, ACS publications, respectively

A metal–organic framework (MOF) is an organic/inorganic hybrid platform that enables the organization of chromophores into a desired arrangement [12]. Chromophores can be hierarchically integrated into a crystalline MOF scaffold, thereby facilitating the study of both short- and long-range energy interactions through crystallographic analysis. Recent works on MOF-based light-harvesting structures have demonstrated their feasibility for energy transfer studies [13]. Hupp and coworkers developed porphyrin-based MOF layers that show significant energy transfer [14, 15]. In a 2013 report, a MOF constructed from zinc-metalated porphyrins showed long-range and anisotropic energy migration (Fig. 2a). The authors attributed the remarkable energy transfer to enhanced pi conjugation in the MOF. Particularly, assigning directionality to the energy transfer is a significant issue in both natural and artificial light harvesting to effectively concentrate the photo-energy in the reaction center.

Inducing a self-assembled complex without the aid of a template has also been used in manufacturing an artificial chromophore assembly. In the case of metal-inserted chromophore molecules, the metal–ligand interaction can form highly stabilized supramolecules with structural integrity. A construction strategy using coordination chemistry for multi-chromophore supramolecular assemblies has been developed as a convenient method to control the intermolecular arrangement [16].

Self-assembly has also received attention in the structural design of an entire antenna complex with a larger scope. For instance, a cyclic architecture of a chromophore assembly can be manufactured. In an approach using self-assembly, several reports have shown that porphyrins can be automatically organized into cyclic architectures of controllable size [17]. Because the cyclic structure of the light-harvesting antenna complex has been considered a key for efficient energy transfer in purple bacteria, the effective energy transfer mechanism on highly symmetric cyclic structures has been studied in several examples. One unique structure of a self-assembled supramolecule is the nanorod-shaped antenna of metal chlorides, first reported by Wurthner and coworkers in 2005 [18]. Recently, Shoji et al. reported nanotube-structured supramolecules of metal chlorophyll derivatives [19]. In this work, magnesium, zinc, and cadmium chlorophyll derivatives synthesized from natural chlorophyll a were self-assembled from a hydrophobic solution into nanotubes 5 nm in diameter (Fig. 2b).

#### Light-harvesting complex of an antenna-reaction center hybrid

The excited energy from the antenna should be collected at the reaction center for electrochemical energy conversion. Thus, the net light-harvesting system is constructed as an integrated hybrid form of an antenna and a reaction center to facilitate cooperative interaction. The configuration of the energy donor and acceptor is the essential factor to drive excitons in the preferred direction.

At the molecular scale, linking molecules covalently stretched from the primary donor to the final acceptor can form an energy bridge. The size and shape of the antenna-reaction center supramolecule can vary from a dyad of a chromophore and an acceptor molecule to a hierarchical dendrimer-shaped complex. Recent examples of light-harvesting systems for excitation energy transfer and conversion are organized in several reviews [20–22].

**Fig. 3:**
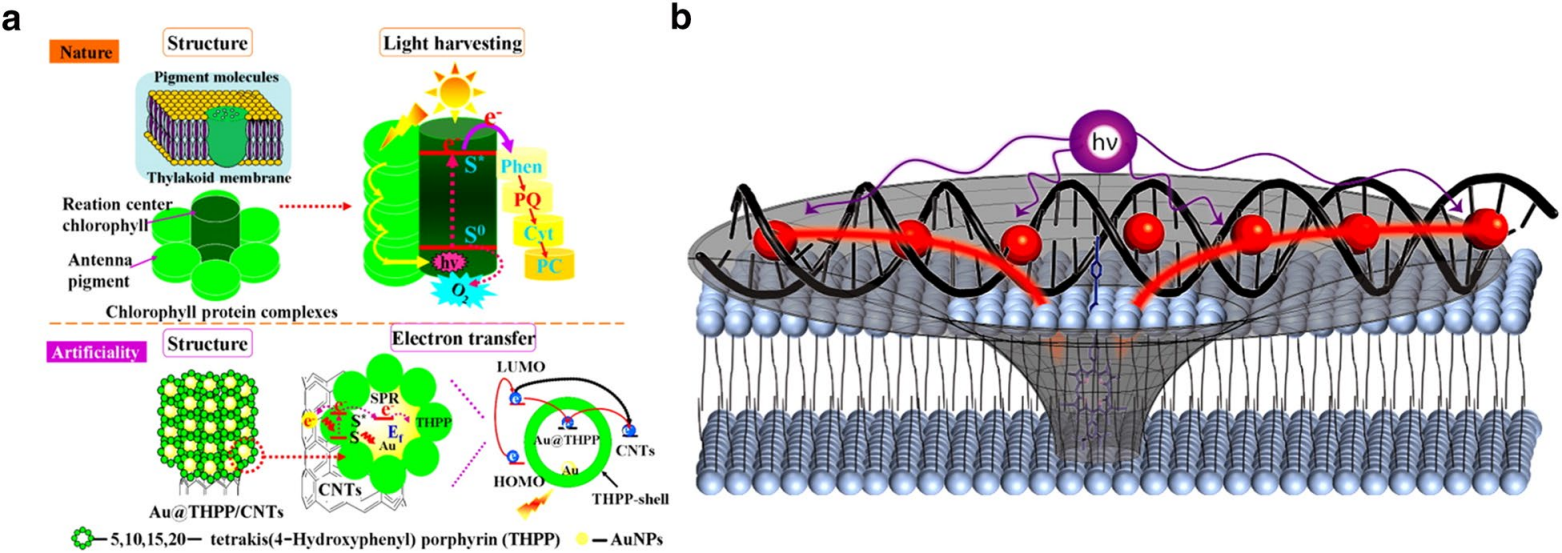
Schematic representation for artificial light harvesting complexes. **a** Porphyrin-based nanohybrid light harvesting complex. **b** DNA-porphyrin assembly for light harvesting Adapted with permission from ref [23] and [24]. Copyright 2016 and 2013, ACS publications, respectively

In more advanced light-harvesting systems analogous to massive photosynthetic proteins, copious chromophores are integrated peripherally oriented to the reaction center. In 2016, Ning et al. reported a porphyrin-based nanohybrid light-harvesting complex using a gold-porphyrin core–shell hybrid aligned on carbon nanotubes [23]. The entire structural design was inspired from the antenna-reaction center cooperated complex embedded within the thylakoid membrane (Fig. 3a). Woller et al. moreover, inserted the accepter into a lipid bilayer and attached donor dyes embedded in a DNA scaffold [24]. In the hybrid system, the DNA-donor complex and acceptor porphyrin play the roles of the antenna and reaction center, respectively, conceptually inspired from the natural light-harvesting system (Fig. 3b). Likewise, massive light-harvesting complexes have been developed by introducing diverse hybrid materials from biomaterials to inorganics.

## Electron transfer and electrochemical energy conversion

The ultimate function of the light reaction of photosynthesis is to convert light energy into chemical energy that can be used in the metabolism of the organism. Following the photo-induced charge separation in the reaction center of the photosystems, the excited electrons move through the photosystem and generate a chemical reductant. The electron-to-chemical energy conversion model has inspired the development of various electrochemical energy devices adapted to man’s demand.

### Electron transport in natural photosynthesis

In oxygenic photosynthesis, the step-wise excitation of electrons occurs in two photosystems derived from light harvesting. The electrons excited from photosystem II (PSII) transfer to photosystem I (PSI) by electron mediators, including quinones and cytochromes, and are re-excited in PSI to be used in the reduction of NADP^+^ into NADPH. The two-step excitation enables the electrochemical energy conversion from only water and mild visible light (680 nm for PSII excitation, 700 nm for PSI excitation) [25]. The electron transport chain is called the Z-scheme, a typical electron pathway comprising step-wise excitation using relatively low energy.

Z-schematic electron transport takes place in the thylakoid membrane, where all photosystems and electron carriers are contiguously inserted. Herein, the adjacent carriers possess a suitable redox potential to accept and pass over the electrons so that they can travel through the long distance of the thylakoid membrane [25]. Because protein has a particular position that accepts and donates electrons, unlike isotropic inorganic materials, the alignment direction of the protein and carrier molecules also affects the electron transfer efficiency.

ATP is another essential source of chemical energy produced via the Z-scheme along with NADPH. While NADP^+^ directly accepts an excited electron via the Z-scheme, ATP synthesis is derived from the proton gradient over the thylakoid membrane generated during the electron transport. Protons are generated during the water oxidation at the Mn cluster of PSII and during the redox electron transfer at plastoquinone [26, 27]. Because the protons are only released to the interior side of membrane, the accumulated protons induce a pH gradient, acidic interior and basic stroma, which is the driving force of proton pumping at ATP synthase [28]. Finally, ATP is synthesized using the proton pumping and utilized as biochemical energy with NADPH in the dark reaction.

### Artificial electron transfer system for energy conversion

#### Photo-electrode for electrochemical reaction

Inspired by the electrochemical energy conversion in the photosystem, an artificial photo-electrode for the redox chemical reaction has been developed. In the artificial inorganic electrode, a semiconductor typically excites electrons up to its band gap in analogy to the photo-excitation of the photosystem. The semiconductor itself, or with the aid of cocatalysts, sequentially catalyzes the desired redox reaction, as the excited electron reduces NADP^+^ at the end of the photosystem. The typical semiconductor photo-electrode utilized in the artificial electrochemical cell includes silicon, hematite, and titanium dioxide. According to their specific energy band positions and catalytic properties, electrodes can be applied for water splitting [29–31], carbon dioxide reduction [32], and other chemical reactions.

As a very direct approach to adapt photosynthetic electrochemical energy conversion, the photosystem protein itself can be used as an electrode material. The photosystem’s modified photo-electrode is promising due to its higher energy conversion efficiency (the charge separation efficiency of PSI is ~100 % [33]) and lower recombination rate compared to that of a semiconductor. However, challenges remain, such as the instability of the photosystems on the electrode and the inefficient transport of photo-electrons from the photosystem to the electrode. Many efforts have been focused on the development of a proper platform to trap the photosystem and transfer charges, and a conducting polymer is often proposed as an electrode material that can fulfill these requirements. Nafion, for example, has been reported for its ability to encapsulate a photosystem while permitting electron transfer [34]. Attributed to its unique selective conductivity for cations, photo-excited electrons can be carried by positively charged electron transfer mediators, such as methyl viologen. On the other hand, a polyaniline-based photo-electrode has also been suggested [35]. Polyaniline can be synthesized on the electrode surface in the form of a three-dimensional network, which can trap photosystems and directly transfer excited electrons through a chain-hopping mechanism.

**Fig. 4:**
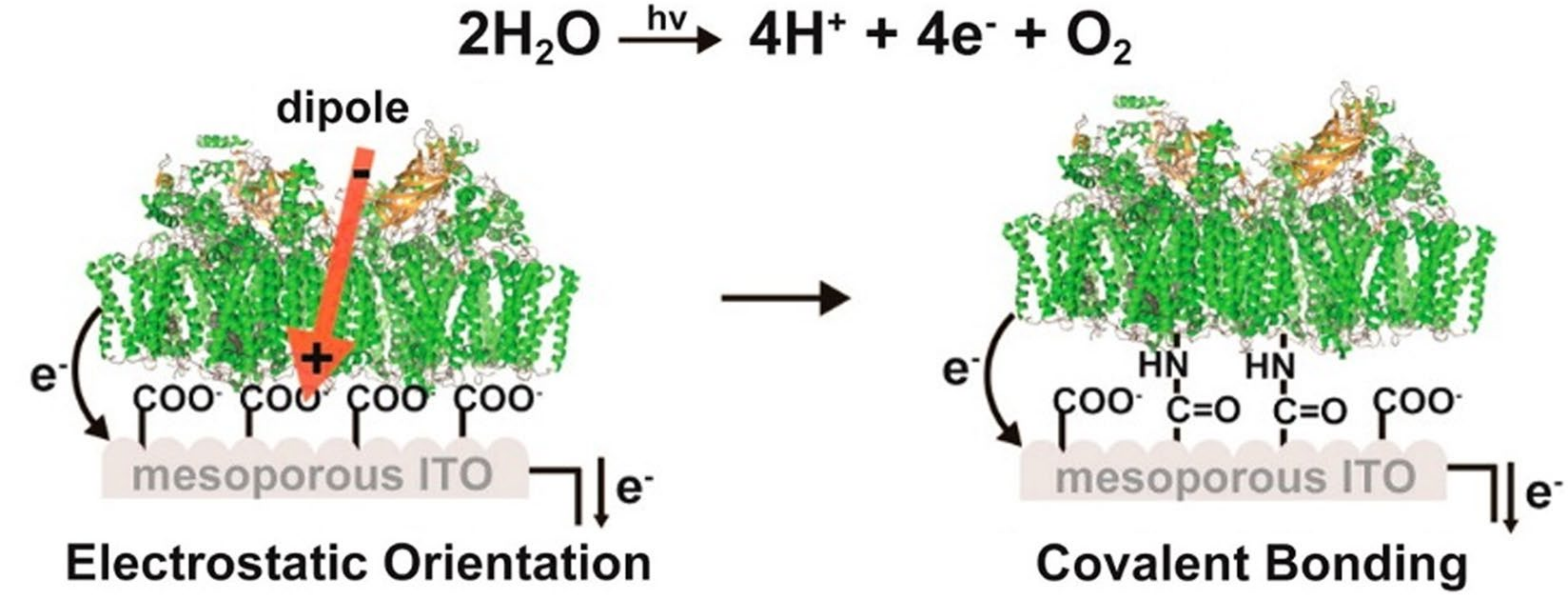
Immobilization of PSII on carboxylated ITO electrode via electrostatic immobilization (*left*) and covalent immobilization (*right*) Adapted with permission from ref [36]. Copyright 2013, ACS publications

Inspired by the directional electron transfer of the photosystem owing to its anisotropic nature, controlling the orientation of the photosystem has been of growing significance in enhancing the efficiency of the photo-electrode. While the electron–hole pair generation in semiconductors is isotropic, the photosystem possesses a precise site of electron emission. Thus, researchers have tried to utilize this feature by aligning photosystems to orient the excited electrons directly toward to the electrode. Kato et al. introduced carboxylic acid functionalized ITO as a supporting electrode for PSII to control the orientation of the protein alignment (Fig. 4) [36]. As the functional group was negatively charged, the positively charged stromal side of PSII was selectively bound to the electrode by electrostatic interaction. This led to direct electron transfer from the electron donor site on the stromal side to the ITO electrode. The linkage was further stabilized by amide bond formation via EDC coupling, which resulted in a greatly enhanced photocurrent and stability of the electrode.

#### Z-schematic fuel production system

Along with the development of artificial photoelectrodes inspired by photosystems, mimics of the full Z-scheme have also been studied. In the artificial Z-scheme, semiconducting materials can replace two photosystems, or proteins can be directly utilized like in the case of photoelectrodes. When proper photoactive materials are selected, the full Z-scheme is completed by introducing a proper electron mediator between the two reaction centers. The final design of the Z-scheme is typically classified into one of two types depending on the electron mediator material: redox ion pairs and electron-conducting metals.

**Fig. 5:**
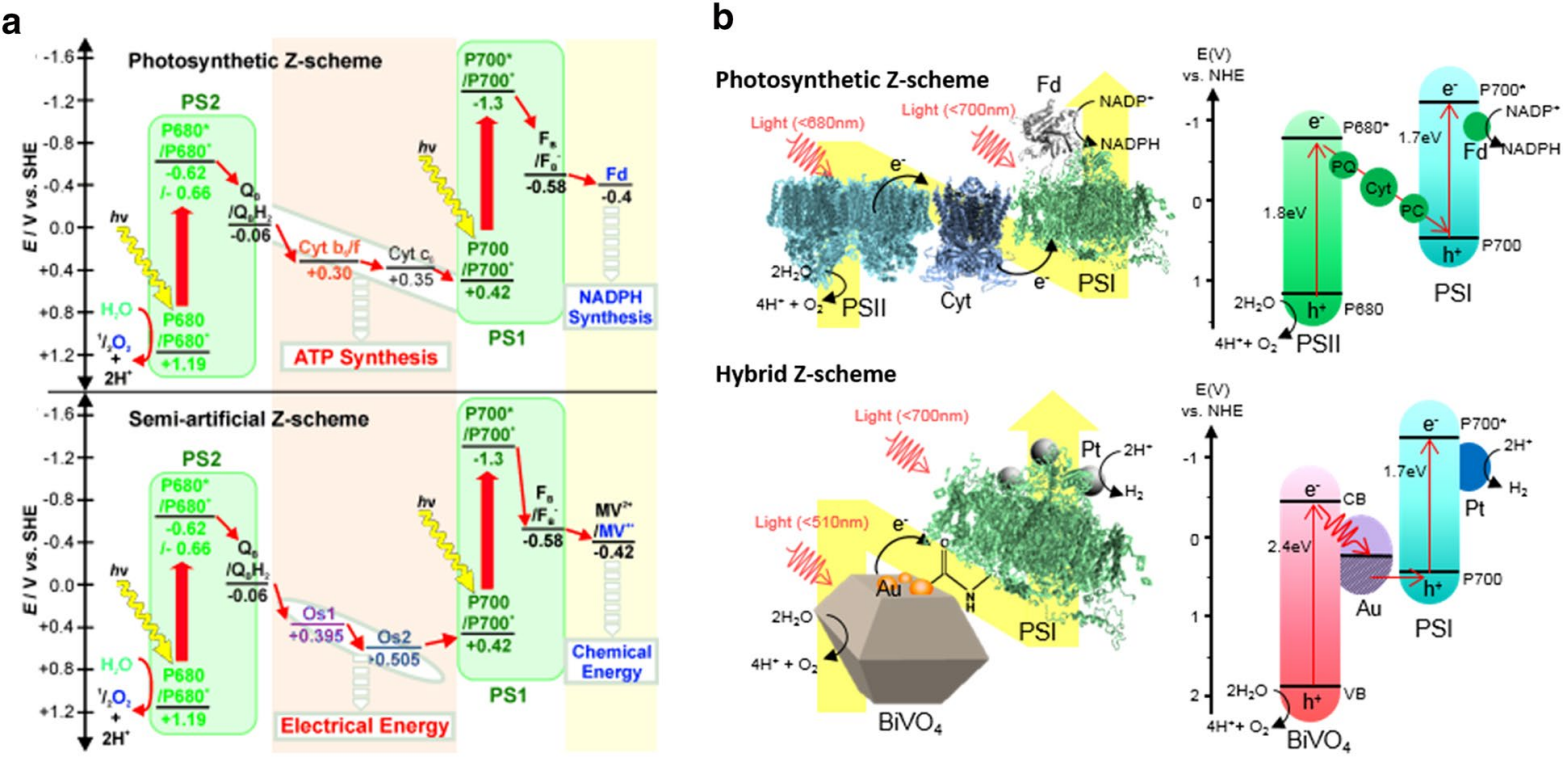
Electron transfer pathway of artificial Z-schematic systems depending on the type of electron mediator. **a** Using redox ion pairs as a mediator. **b** Using electron conducting metals as a mediator Adapted with permission from ref [38] and [40]. Copyright 2013 and 2015, WILEY–VCH Verlag GmbH & Co. KGaA, Weinheim, respectively

When redox ion pairs are applied as electron mediators in the artificial Z-scheme, the two reaction centers are physically divided and interact via the redox reaction of the ions. Electron loss resulting from the unintended reverse reaction and the narrow pH window are typical drawbacks of the system that limit the electron transfer efficiency [37]. The Rogner group selected an Os-based redox hydrogel as an electron mediator and designed a serially connected bio-photovoltaic cell by using PSI and PSII as photoelectrodes (Fig. 5a) [38]. The system also mimicked the ATP synthesis process of the Z-scheme along with electron transfer, generating light to electrical energy conversion driven by the potential difference in the Z-schematic electron flow. Furthermore, the photovoltaic properties, such as power output and energy conversion efficiency, can also be enhanced by tuning the redox potential of each electron mediator for the two photoelectrodes [39].

Electron-conducting metals are also utilized as electron mediators, providing a direct electron pathway between the two reaction centers. In contrast to redox ion pairs, two reaction centers can be physically combined by ohmic contact to form an all-solid-state system. Thus, the electron loss from the reverse reaction can be hampered, and a wide pH window can be achieved [37]. All-solid-state has been typically applied in designing the Z-scheme of two semiconductors. However, in 2015, our group developed a hybrid Z-scheme of a photosystem and a semiconductor in the form of an all-solid-state Z-scheme for the first time [40]. The work also has significance in demonstrating the first photosystem-based Z-scheme for hydrogen evolution from water. BiVO_4_ has been used as a water-oxidizing reaction center, analogous to PSII in the natural Z-scheme, considering its proper band gap for charge separation and electron transfer to PSI (Fig. 5b). Moreover, inspired by the directional electron emission in photosystems, the electron-mediating metals were selectively deposited on electron-accumulated {010} BiVO_4_ facets to facilitate anisotropic electron transfer.

## Carbon fixation

Carbon fuel is the ultimate practical energy source for both living organisms and machine engines. Nature uses carbon dioxide as an infinite carbon source and produces sugar via the photosynthetic dark reaction. On the other hand, man uses coal from underneath the ground, which has been rapidly depleted after industrialization and is expected to lead to a drastic carbon energy deficiency soon. Research on the development of a novel carbon cycle inspired by nature has been performed to secure a stable route for energy production.

### Dark reaction in natural photosynthesis

Concurrently with the light reaction in the thylakoid membrane, the dark reaction proceeds in the stromal region. As it directly consumes the products of the light reaction (NADPH and ATP), it also occurs during the daytime, although it is called the ‘dark’ reaction. The net reaction consists of three cyclic sequential steps: carbon fixation, reduction and regeneration of ribulose. Overall, it is called the ‘Calvin cycle’, in which three carbon dioxide and five water molecules are converted into a 3-carbon sugar, a half molecule of glucose (3CO_2_ + 6NADPH + 5H_2_O + 9ATP → glyceraldehyde-3-phosphate (G3P) + 2H^+^ + 6NADP + + 9ADP + 8P_i_).

Carbon fixation is the first process in the Calvin cycle, in which the carboxylation of a 5-carbon compound, ribulose, into a 6-carbon compound proceeds by the capture of CO_2_. Herein, an enzyme called rubisco facilitates CO_2_ binding and induces carboxylation at its active site [41]. The 6-carbon product then immediately splits into two 3-carbon compounds due to its structural instability and is chemically reduced by ATP and NADPH in the next step. Finally, the reduced form of the carbons is regenerated into ribulose, the 5-carbon starting compound for carbon fixation, through several chemical synthetic processes, which completes the cycle.

The fixation of CO_2_ by rubisco is indispensable for the production of almost every form of bioenergy, but at the same time, it is the limiting step in the Calvin cycle. Rubisco is one of the most abundant enzymes existing on earth and is the primary contact point for fixing inorganic carbon. Despite its significant function, the efficiency is low, limiting that of the net cycle. Because its active site also accepts oxygen as a substrate and catalyzes photorespiration, the net carboxylation is suppressed [41, 42]. Thus, the activity is highly sensitive to the cellular gas concentration and is also regulated by other factors including temperature, water stress, and ion concentration.

### Electrochemical carbon fixation

Inspired by natural photosynthesis, many studies have been conducted on utilizing carbon dioxide in fuels [43–48]. One of the main approaches is to replace rubisco with other efficient catalysts. By substituting the enzyme with artificially designed catalysts, the overall efficiency of the carbon fixation can be largely improved.

Recently, the electrochemical conversion of carbon dioxide has gained much attention as an artificially developed carbon fixation system [47, 49–51]. This is because it can be operated by renewable energy in moderate conditions such as at room temperature and easily manipulated for scale-up in industry. In the electrochemical reduction of carbon dioxide, the gas dissolved in the electrolyte reacts with protons and electrons under an electrical potential and is finally converted into products such as carbon monoxide, formate, and methanol. This method operates in a similar manner to natural photosynthesis, as both of them require applied energy, protons and catalysts in moderate conditions. Therefore, this section will focus mainly on the electrochemical conversion of carbon dioxide into other carbon fuels.

One approach is to electrochemically reduce carbon dioxide using a homogeneous catalyst. Homogeneous catalysts refer to catalytically active molecules homogeneously dissolved in an electrolyte. A great deal of molecules have been explored as electrocatalysts for carbon fixation [52–59]. Most of them involve transition metals such as Pd [55, 56], Ru [57] and Re [54, 58, 59], which are too expensive to be used frequently. Additionally, these systems exhibit low reactivity and a short lifetime due to their chemically unstable nature.

**Fig. 6:**
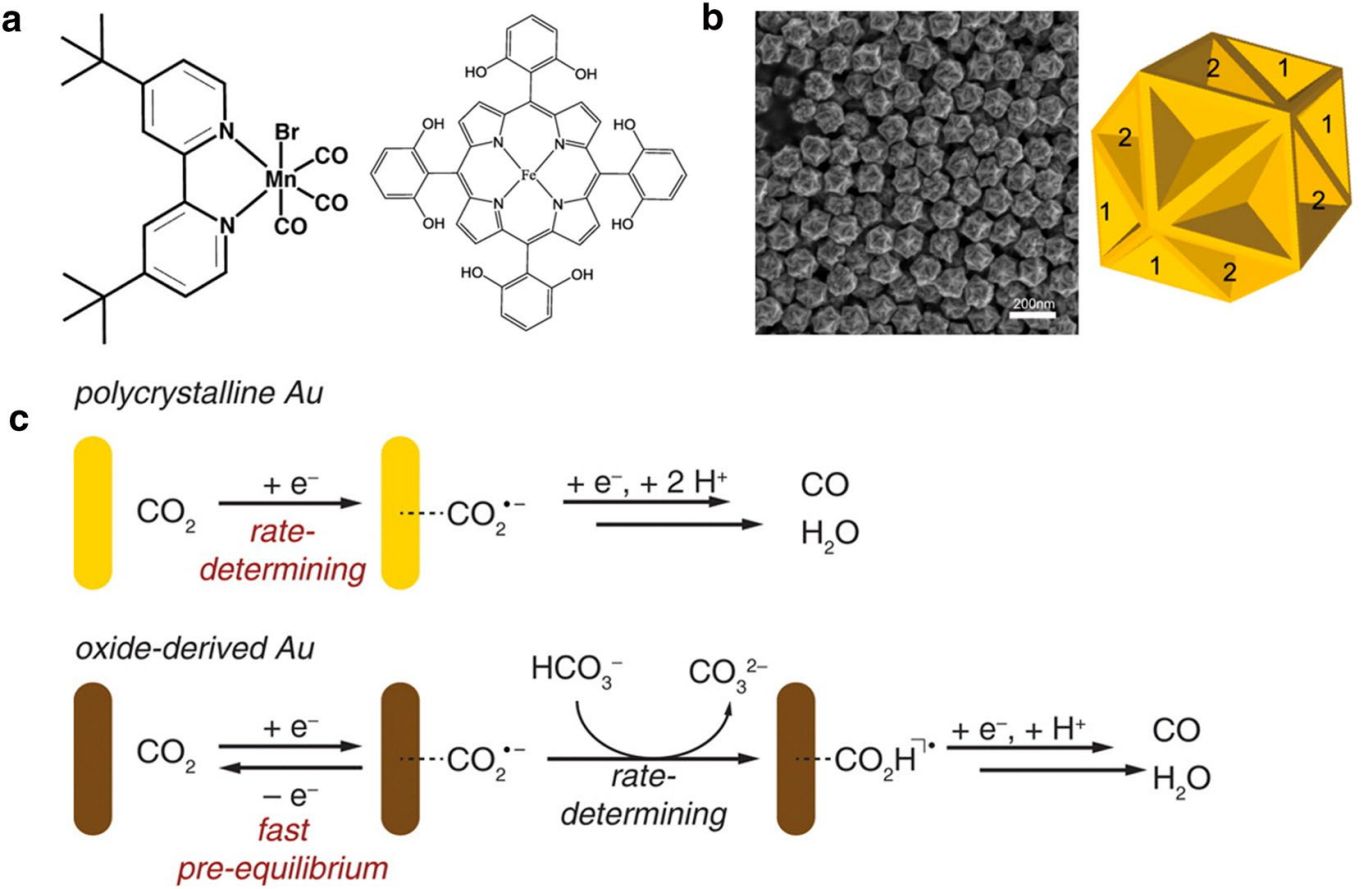
**a** Homogeneous catalysts for the reduction of carbon dioxide. (*left*) Mn(bpy-tBu)(CO)_3_Br and (*right*) Iron 5,10,15,20-tetrakis(2′,6′-dihydroxylphenyl)-porphyrin. Adapted with permission from ref [61] and [62]. Copyright 2013, ACS publications and 2013 RSC Publishing, respectively. **b** Morphology of concave RD nanoparticles. SEM image (*left*) and the corresponding model (*right*). Adapted with permission from ref [70]. Copyright 2015, ACS publications. **c** Mechanistic model for the reduction of carbon dioxide to carbon monoxide on polycrystalline Au and oxide-derived Au. Adapted with permission from ref [72]. Copyright 2012, ACS publications

There have been a number of studies performed to overcome these disadvantages of homogeneous catalysts. The Kubiak group studied the homogeneous catalysis of carbon dioxide using bipyridine carbonyl catalysts with metal ions [58, 60, 61]. When they substituted for the rhenium in a Re(bpy-R)(CO)_3_X scaffold with manganese (Fig. 6a), which is more abundant, their new catalysts exhibited activity toward carbon dioxide reduction with the addition of Brönsted acids [61]. Mn(bpy-*t*Bu)(CO)_3_Br operated at a lower overpotential compared to Re catalysts with great Faradaic efficiency to produce carbon monoxide. The Mn catalysts did not show catalytic activity without weak Brönsted acids, which may be attributed to the protonation of a M-CO_2_ adduct and the eventual activation of the reduction process.

Savéant and co-workers investigated catalysts based on metalloporphyrins [49, 62]. They focused on iron(0) tetraphenylporphyrins with various functional groups as shown in Fig. 6a. Similar to the above-mentioned observation by the Kubiak group, the Savéant group also revealed that a local proton source enhances the catalytic activity of iron(0) porphyrins [62]. Additionally, they discovered that the presence of phenolic OH groups accelerates the catalytic reaction because of the resulting high local concentration of protons.

Another approach is to design heterogeneous catalysts that are active for the reduction of carbon dioxide. Heterogeneous catalysts include not only electrodes with catalytic molecules deposited on them but also electrodes themselves. Generally, transition metals that are known to reduce carbon dioxide, such as Au [63, 64], Ag [65, 66] or Cu [67–69], have been used as electrodes. Despite their natural ability to catalyze the carbon dioxide reduction, a large number of studies have been devoted to improving their reactivity.

Recently, our group demonstrated that Au nanoparticles with a concave rhombic dodecahedral (concave RD) shape can show superior electrocatalytic activity for the conversion of carbon dioxide to carbon monoxide (Fig. 6b) [70]. Nanoparticles were synthesized by using 4-aminothiophenol as a shape modifier and were drop-casted on carbon paper for the evaluation of their electrochemical performance. HRTEM images showed that the Au nanoparticles contained various high-index facets such as (331), (221), and (553). The prepared electrode exhibited improved reactivity and selectivity toward the electroreduction of carbon dioxide compared to typical polycrystalline Au electrodes, a known catalyst for the production of carbon monoxide from carbon dioxide. Thus, it has been proposed that the high activity of the concave RD was achieved by the higher-index facets on its surface.

The Kanan group introduced oxide-derived metal electrodes, which have a high catalytic ability for the reduction of carbon dioxide [71, 72]. In 2012, they first reported an oxide-derived Au electrode that was fabricated by electrochemically reducing a previously oxidized Au electrode [72]. The resulting electrode showed the formation of Au nanoparticles and a highly selective activity to reduce carbon dioxide into carbon monoxide with a very low overpotential. Although the group proposed that the oxide-derived Au electrode stabilizes CO_2_^−^ better than polycrystalline Au, a clear mechanistic understanding is still not available (Fig. 6c).

Despite the recent development of various catalysts, the current technologies of the carbon dioxide electrochemical reduction have unresolved challenges. These challenges include low activity, product selectivity, and stability, which make it insufficient for practical application. In addition, unlike natural photosynthesis, in which C_3_ products are formed after one Calvin cycle, artificial photosynthesis seems to produce mostly C_1_ products. This trend directly implies the current status that most of the artificial electrocatalysts are not suitable for C–C coupling between C_1_ products. Therefore, extensive work is still required to develop new types of catalysts that can overcome these challenges and produce high-carbon fuels.

## Conclusions

In this review, recent developments in energy harvesting, transfer and conversion systems inspired by natural photosynthesis have been introduced, focused on strategies to take scientific lessons from nature. In photosynthesis, three serial processes consisting of light harvesting in the antenna, Z-schematic electron transfer in the thylakoid membrane, and carbon fixation via the dark reaction operate collaboratively to convert light into electrochemical energy. Numerous artificial systems have been inspired from each process, as presented in the review. The photosynthetic protein itself is an interesting model to adapt in new electrochemical systems due to its anisotropic nature and unique catalytic properties. However, to overcome its instability and tune its catalytic function to other practical energy production, the further engineering of the protein or hybridization with other organic/inorganic materials is necessary. Additionally, as the surrounding environment of the photosystem affects the overall operation, controlling the peripheral conditions of the net artificial system is also a key requisite, such as by forming vesicles or confining the materials in gels. Finally, inspired by the ultimate production of high carbon fuels in photosynthesis, higher value added carbon fuel products should be considered as the ultimate goal in mimicking photosynthesis.
